# Effect of Concentrated Fibroblast-Conditioned Media on *In Vitro* Maintenance of Rat Primary Hepatocyte

**DOI:** 10.1371/journal.pone.0148846

**Published:** 2016-02-10

**Authors:** Dayeong Jeong, Chungmin Han, Inhye Kang, Hyun Taek Park, Jiyoon Kim, Hayoung Ryu, Yong Song Gho, Jaesung Park

**Affiliations:** 1 School of Interdisciplinary Bioscience and Bioengineering, Pohang University of Science and Technology (POSTECH), Pohang, Gyeong-buk, Republic of Korea; 2 Department of Mechanical Engineering, Pohang University of Science and Technology (POSTECH), Pohang, Gyeong-buk, Republic of Korea; 3 Department of Life Science and Division of Molecular and Life Sciences, Pohang University of Science and Technology (POSTECH), Pohang, Gyeong-buk, Republic of Korea; 4 Chadwick International School, Songdo, Incheon, Republic of Korea; University of Tampere, FINLAND

## Abstract

The effects of concentrated fibroblast-conditioned media were tested to determine whether hepatocyte function can be maintained without direct contact between hepatocytes and fibroblasts. Primary rat hepatocytes cultured with a concentrated conditioned media of NIH-3T3 J2 cell line (final concentration of 55 mg/ml) showed significantly improved survival and functions (albumin and urea) compared to those of control groups. They also showed higher expression levels of mRNA, albumin and tyrosine aminotransferase compared to hepatocyte monoculture. The results suggest that culture with concentrated fibroblast-conditioned media could be an easy method for *in vitro* maintenance of primary hepatocytes. They also could be contribute to understand and analyze co-culture condition of hepatocyte with stroma cells.

## Introduction

Cell-cell interaction in liver tissues is essential for hepatic function and regeneration [1–3]. In hepatic tissue engineering, interactions between parenchymal cells and non-parenchymal cells are particularly important. Many biological molecules mediate communication in hepatic tissue; these include as numerous cytokines, extracellular matrices (ECMs), and chemokines that mediate endocrine, paracrine and autocrine signaling. For example, ECM proteins bind to integrins, thereby activating signaling pathways that regulate morphology, adhesion, migration, and proliferation of cells. Fibroblasts that are co-cultured with hepatocytes secrete ECMs, cytokines and growth factors, so metabolic activities of hepatocyte in co-culture with fibroblast remain elevated longer than those of hepatocytes in single cultures [4,5]. In addition, unlike other tissues, direct transfer of small molecules through gap junctions is considered critical for hepatic functions, but little is known about these interactions [6].

Primary hepatocytes have been widely used in studies of drug metabolism, toxicology and bio-artificial livers [7–11]. However, because they rapidly lose their functions during *in vitro* culture, *in vitro* maintenance of primary hepatocytes has been an important challenge in many hepatocyte-related studies [12–14]. When cultivated with other non-parenchymal cell types *in vitro*, hepatocytes can maintain viability and functions for several weeks [15–17]. Various biological molecules, like receptors, soluble growth factors and ECM, have been investigated as possible mediators of this co-culture effect. In spite of many studies, the mechanism by which it improves hepatocyte function remains unknown. Some studies suggested that direct contact between hepatocytes and stromal cells can make hepatocyte improve viability and function for at least a limited time [18–20]. However, in a study that used a trans-well insert, in which hepatocytes were seeded on a 0.45-μm-pore filter, and stroma endothelial cells were seeded in the underlying well, hepatocytes secreted more albumin than did cells in the control co-culture in which hepatocytes were in direct contact with stroma cell [16,21]; this result suggests that the influence of the stromal cells is mediated by chemical molecules rather than by physical contact. The other method for in-direct contact culture is to use conditioned media to maintain hepatic functions. The conditioned media of stroma cells showed a slight effect on maintenance of hepatocyte viability and function [22]; this result suggests that soluble factors alone have a limited effect, and therefore that direct contact is necessary in hepatic function maintenance [23]. In contrast, hepatocytes embedded in collagen gel and treated with conditioned medium showed similar levels of albumin secretion as those in co-culture condition with direct contact. Therefore, to improve understanding of how stroma cells affect hepatocyte functions, detailed research is required [24].

In this report, the effects of concentrated fibroblast-conditioned media (F-CM) were tested to determine whether hepatocyte function can be maintained without direct contact between the cells. We confirmed that primary rat hepatocytes cultured with concentrated F-CM of NIH-3T3 J2 cell line showed significantly improved survival and functions (albumin and urea synthesis) compared to non-treated control groups. Gene expression levels of hepatocyte specific genes increased more in the hepatocyte culture with F-CM than in the hepatocyte monoculture. The effect of concentrated F-CM was reconfirmed by treating the hepatocytes with different concentrations of F-CMs; the result showed a dosage dependent-improvement of hepatic function. Therefore, we concluded that the contact between hepatocytes and stroma is not always required; i.e., that treatment with fibroblast-secreted factors is sufficient to maintain *in vitro* hepatic function when the concentration of the factors is sufficient. Thus, this new method of hepatocyte culture would improve the efficiency of related studies.

## Materials and Methods

**Fig 1:**
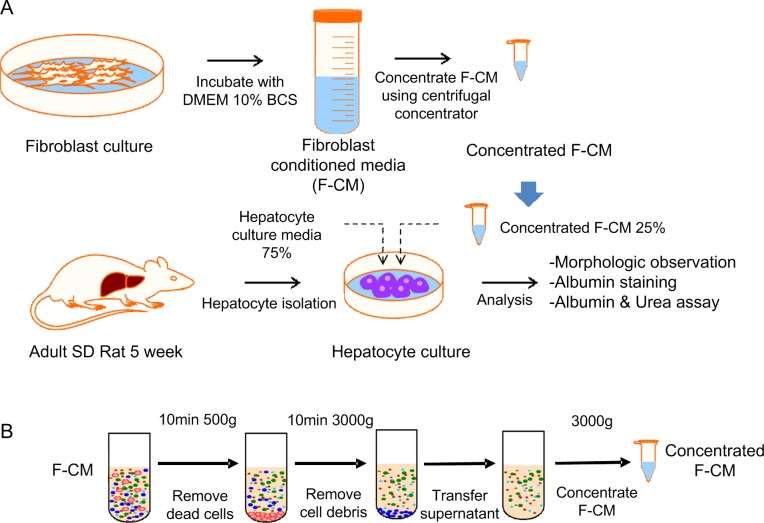
Procedure for preparation of concentrated F-CM. (A). Fibroblasts were rinsed with PBS and refreshed with DMEM supplemented with 10% depleted BCS media. The cells were incubated at 37°C for 24 h to condition the media with fibroblasts. The media were then collected and pre-cleaned by serial centrifugation and further concentrated using centrifugal concentrator units (3-kD Centripreps). Isolated hepatocytes were cultured with a medium that consisted of 75% normal hepatocyte culture media and 25% F-CM (final F-CM concentration: 55 mg/ml). (B) Conditioned medium was pre-cleaned by serial centrifugation. First, the medium was centrifuged at 500 x g for 10 min to remove cells. Supernatants were centrifuged again at 3000 x g for 20 min to remove cell debris and then concentrated by a factor of ~80 using 3-kD Centripreps.

The experiment consisted of preparation of hepatocytes and fibroblasts, treatment of conditioned media, and several assays (Fig 1A and 1B).

### Ethics Statement

All animal experiments were planned to minimize animal suffering, and were approved by the Institutional Animal Care and Use Committee at POSTECH, Pohang, Republic of Korea (approval number: 2013-01-0016R1).

### Hepatocyte isolation and culture

Hepatocytes were isolated from 5-week-old female Sprague-Dawley (SD) rats (Central Lab. Animal Inc.) weighing 150 to 200 g. The SD rat was anesthetized using ether, and the abdomen was cut using scissors. The stomach and entrails were moved to the right to reveal the portal vein. A catheter was inserted into the portal vein and Kreps-Ringer Bicarbonate buffer (KRB, Sigma) with 1.5 mM EDTA buffer was perfused. When blood had completely drained from the liver, 0.05% collagenase (Sigma) with KRB solution was perfused until the liver tissue was digested. The digested liver tissue was separated into a petri dish with KRB, and hepatocytes in the tissue were harvested. The cell suspension solution was washed twice using a centrifuge at 70 g for 5 min. To isolate living hepatocytes, the cell pellet was suspended in KRB and was mixed it with Percoll (Sigma) and 10x HPSS solution and the mixed solution was centrifuged at 70 g for 20 min. The cell pellet was suspended in KRB and washed using centrifuge at 70 g for 5 min, then primary hepatocytes were isolated. The procedure yielded 100~300 million hepatocytes with viability > 95% measured using Trypan Blue exclusion. Tissue-culture dishes (35 mm) were coated with type-1 rat tail collagen (0.125 mg/ml in distilled water, BD) for 30 min at 37°C. The collagen solution for coating was discarded, then hepatocytes were seeded into hepatocyte culture medium and incubated in 90% air/10% CO_2_ at 37°C. The hepatocyte culture media consisted of DMEM (Hyclone) supplemented with 10% fetal bovine serum (Hyclone), 7 ng/ml glucagon (Bedford Laboratories), 7.5 μg/ml hydrocortisone (Sigma), 0.5 unit/ml insulin (Sigma), 20 ng/ml epidermal growth factor (Sigma), 200 unit/ml penicillin, and 200 μg/ml streptomycin (Gibco).

NIH-3T3 J2 fibroblast was selected as a stroma cell for co-cultures because hepatocytes co-cultured with these cells have enhanced proliferation and maintenance of functions (e.g., albumin synthesis) compared to those co-cultured with other stromal cells.[2,25,26] NIH-3T3 J2 was obtained from American Type Culture Collection (ATCC). NIH-3T3 J2 fibroblasts were seeded at 3:1 (fibroblasts: hepatocytes) density on the day after hepatocyte seeding, because previous references suggested that this hepatocyte-to-fibroblast ratio would yield the best results.[27,28] The total initial cell number (hepatocytes + fibroblasts) was 1.4 x 10^6^ cells per 6-well plate dish. Culture medium was changed daily and the samples were collected and stored at 4°C until further hepatic function analysis.

### Fibroblast culture and conditioned medium preparation

NIH-3T3 J2 fibroblasts (ATCC) were cultured in a 150-mm tissue-culture dish at 37°C in a humidified atmosphere of 90% air/10% CO_2_. The culture was maintained in DMEM supplemented with 10% bovine serum (BCS, Gibco). When fibroblast culture became confluent, cells were rinsed with PBS and the growth medium was replaced with DMEM supplemented with 10% depleted BCS. The culture was then maintained for 24 h to condition the medium. After 24 h, the medium was collected and centrifuged at 500 x g for 10 min then at 3,000 x g for 20 min to remove cell debris. The pre-cleaned medium was then concentrated from ~120 ml initial volume to ~1.5 ml final volume using Centriprep YM-3 centrifugal units (Millipore).

### Treatment of conditioned media for primary hepatocyte culture

The amount of proteins in the concentrated conditioned medium was determined using a Bradford protein assay. The protein concentration of prepared media was then adjusted to 220 mg/ml for further experiments. The prepared stock solution was then mixed with fresh hepatocyte culture media at a volume ratio of 1:3 (concentrated conditioned medium: hepatocyte media) for further hepatocyte cultures. Different dilutions of conditioned media were prepared by mixing the media in different volume ratios. The media of the hepatocyte cultures were changed daily, and the morphology of samples was observed under phase-contrast microscope (OLYMPUS).

### Albumin immunofluorescence staining

Hepatocyte cultures in 6-well plate dishes were rinsed twice with PBS, fixed in 4% paraformaldehyde (Sigma) at room temperature (RT) for 20 min, then washed three times with PBS. Then 0.1% Triton X-100 (Sigma) in PBS was added to each plate for 10 min to permeabilize cells. The cells were washed three times with PBS, incubated with blocking buffer (1% BSA in PBS) for 1 h at RT. The cells were washed 3 times with PBS, then stained for 1 h at RT with sheep anti-rat albumin polyclonal IgG antibody (1:1000 dilution, A110-134A, Bethyl Laboratories, Inc). The cells were washed again three times with PBS, then stained with Alexa Fluor 488 conjugated donkey anti-sheep IgG polyclonal secondary antibody (1:1000 dilution, A11015, Invitrogen) for 1 h at RT. Stained samples were washed three times with PBS and observed under a fluorescence microscope (OLYMPUS)

### Reverse transcription-polymerase chain reaction

Reverse transcription-polymerase chain reaction (PCR) was conducted to check gene expression of the hepatocyte, NIH-3T3 J2 fibroblast, and three samples which were hepatocyte monoculture, hepatocyte co-culture with NIH-3T3 J2 and hepatocyte monoculture with F-CM. Hepatocyte and NIH-3T3 J2 were regarded as positive and negative control. As soon as hepatocytes were isolated from rat, mRNA of hepatocytes was isolated. mRNA of three samples collected on day 7 were isolated with Trireagent^®^ (Sigma) and chloroform (sigma) according to manufacturer’s manual. The isolated mRNA (1000 ng) was reverse transcribed to cDNA. The specific cDNA strands according to each primer were amplified using a polymerase chain reaction kit (Promega)

The following primers were used:

β-actin: Forward AGCCATGTACGTAGCCATCC

          Reverse CTCTCAGCTGTGGTGGTGAA

Albumin: Forward CTCTCGCTGAGCTGGTGAAA

          Reverse CAGCCTTGCAACACTTGTCC

TAT: Forward AAGTCCAATGCGGACCTCTG

          Reverse TCAACCGCTCTGTGAACTCC

The process of reaction consisted of denaturation (94°C for 5 min), 30~35 cycles of amplification (94°C for 30 s, 55°C for 30 s, 72°C for 1 min), and extension (72°C for 10 min). The reaction product were separated on a 1.5% agarose gel which was stained with SYBR^®^Green (molecular probe). The images were taken using a BioDoc-It imaging system (UVP).

### Quantitative polymerase chain reaction

The cDNAs which were amplified by PCR (previously mentioned) were used in quantitative PCR (qPCR) using a iQ SYBR Green Supermix (Biorad). The amplification of the cDNA was conducted in 35 cycles of: 1) heating at 94°C for 10 min, 2) at 95°C for 10 s, 55°C for 10 s and 72°C for 20 s, and 3) 72°C for 10 min using a iCycler iQ^tm^ Real-Time PCR Detection System (Biorad). The comparative Ct method was used for relative measurement of gene expression level against β-actin gene.[29]

### Albumin ELISA

Cultured media samples were collected every two days for albumin quantification. For ELISA, 96-well plates were first coated with 0.1 mg/ml rat albumin (EMD Millipore) in coating buffer overnight at 4°C. Then the albumin coated plates were washed with 0.05% Tween-20 (Sigma) in PBS (washing buffer). The samples and standard were loaded into wells and HRP-conjugated sheep anti-rat albumin antibody (1:10000, A110-134P, Bethyl Laboratories, Inc.) was added with 1% BSA in PBS. The samples were incubated for 1.5 h at 37°C. The cells were washed three times with PBS, then 100 μl of *o*-phenylenediamine solution (OPD tablet, Sigma) was added to each well. After 5 min incubation, 50 μl of 8 N aqueous sulfuric acid (Sigma) was added to inhibit the reaction. Finally the plate was read on a microplate reader (Molecular Devices) at 450 nm.

### Urea assay

Cultured media samples were collected every two days for urea quantification using a commercially-available assay kit (StanBio). First, 10-μl samples and standards were loaded in a 96-well plate. Loaded wells were then mixed and incubated with 100 μl of enzyme reagent for 10 min at RT; this enzyme degrades urea to into ammonia and carbon dioxide. Samples were then mixed with 100 μl color reagent and incubated for 10 min at RT. Absorbance was read using a microplate reader (Molecular Devices) at 600 nm.

### Size-exclusion chromatography using HPLC system

Media (120 ml) in which NIH-3T3 J2 had or had not been cultured were concentrated to 1.5 ml (~ 80 fold) as described above. Concentrated samples were then ultra-centrifuged at 100,000 x g for 1 h to remove heavy proteins, then the supernatant was stored for use in size-exclusion chromatography (SEC). Chicken ovalbumin (45 kDa), ribonuclease A (13.7 kDa) and insulin (5.8 kDa) were used as standards for estimating protein size. A Bio-Sil SEC 250–5 column (300 x 7.8 mm, Bio-Rad) was washed with filtered deionized water. Before chromatography was performed, the sample was centrifuged again at 12,000 x g for 5 min to remove any aggregates that could clog the column. Then a 50-μl sample was analyzed using an HPLC system (SHIMADZU) running at 0.2 ml/min flow rate for 80 min.

An additional SEC analysis was performed using concentrated cultured serum free (SF) media and concentrated SF media (supporting information). A Bio-Sil SEC 250–5 column (300 x 7.8 mm, Bio-Rad) was washed and used for mobile phase with filtered 0.1 M sodium phosphate buffer (pH 7.0). Before chromatography was performed, the sample was centrifuged again at 12,000 x g for 10 min to remove any aggregates that could clog the column. Then 20-μl of each sample was analyzed using an HPLC system (SHIMADZU) running at 0.5 ml/min flow rate for 50 min.

### Statistical analysis

Experiments were repeated three times with triplicate samples for each condition. Data were first tested for normality by using the Shapiro-Wilk test and for homogeneity of variance by using Levene’s test, with p>0.05 considered normally distributed and equal variances respectively. Statistical analysis was performed using one way ANOVA and *post hoc* Tukey’s pair-wise comparisons with 95% confidence level. All statistical analyses were conducted using open-source R software (version 3.1.2, http://www.R-project.org).

## Result

Fibroblast media were concentrated using a 3-kDa membrane, so molecules > 3 kDa may be enriched in the final solutions. The resulting concentrated F-CM was added (final concentration of 55 mg/ml) to hepatocytes that had been cultured on collagen-coated dishes. Hepatocyte monoculture, and hepatocytes co-cultured with 3T3-J2 fibroblasts were also maintained for use in hepatic function comparisons. The effect of F-CM on maintenance of morphology and functional characteristics of hepatocytes was evaluated.

**Fig 2:**
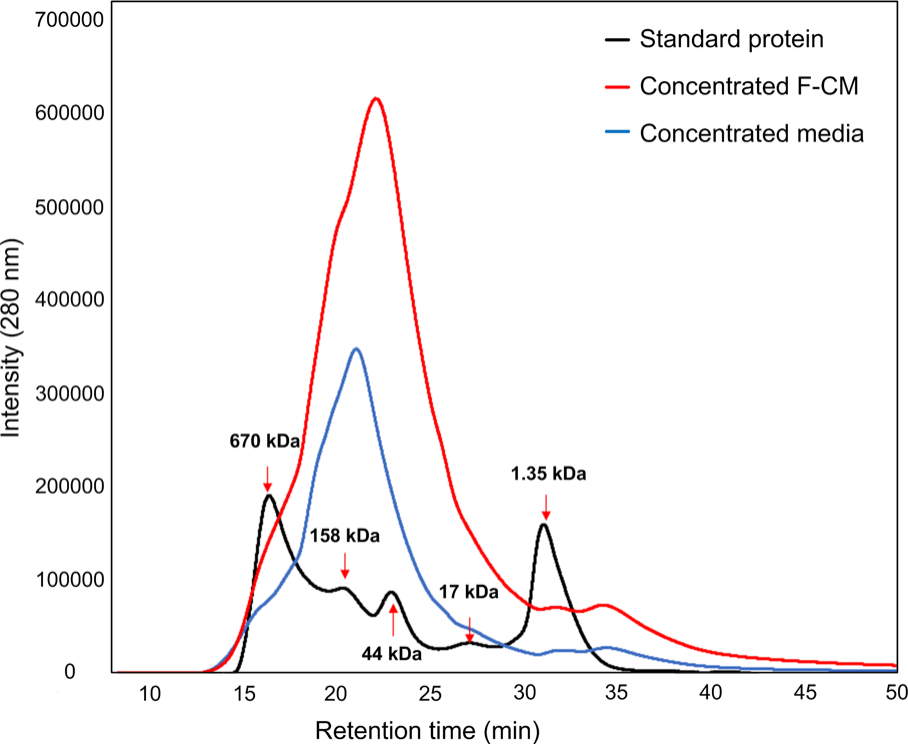
Size exclusion chromatography results of cultured F-CM and non-cultured F-CM. Qualitative composition of concentrated F-CM (red line) and concentrated medium (unconditioned, blue line) were both analyzed using size exclusion chromatography. Contents > 50 kDa were removed before analysis to prevent clogging of columns. Black arrows: reference sizes.

Before the F-CM was applied to hepatocyte cultures, its characteristics were assessed. To verify that rat albumin and urea were only produced by hepatocytes, not by fibroblasts, the concentrations of albumin and urea in fresh F-CMs were quantified. Measured concentrations of rat albumin and urea in fresh F-CM that might have been secreted by fibroblasts were almost negligible (rat albumin: concentrated F-CM 0.788 μg/ml, concentrated DMEM 10% BCS 0.082 μg/ml; urea: concentrated F-CM 6.66 μg/ml, concentrated DMEM 10% BCS 8.78 μg/ml). Therefore, the measured rat albumin and urea values in subsequent sections would have been mainly affected by the function of cultured hepatocytes. The qualitative changes in contents of concentrated F-CM were also assessed using SEC; notable changes in protein composition were detected as peaks appeared after about 30 min (~45 kDa), 40 min (>13.7 kDa) and 60 min (>5.8 kDa) retention time. (Fig 2).

**Fig 3:**
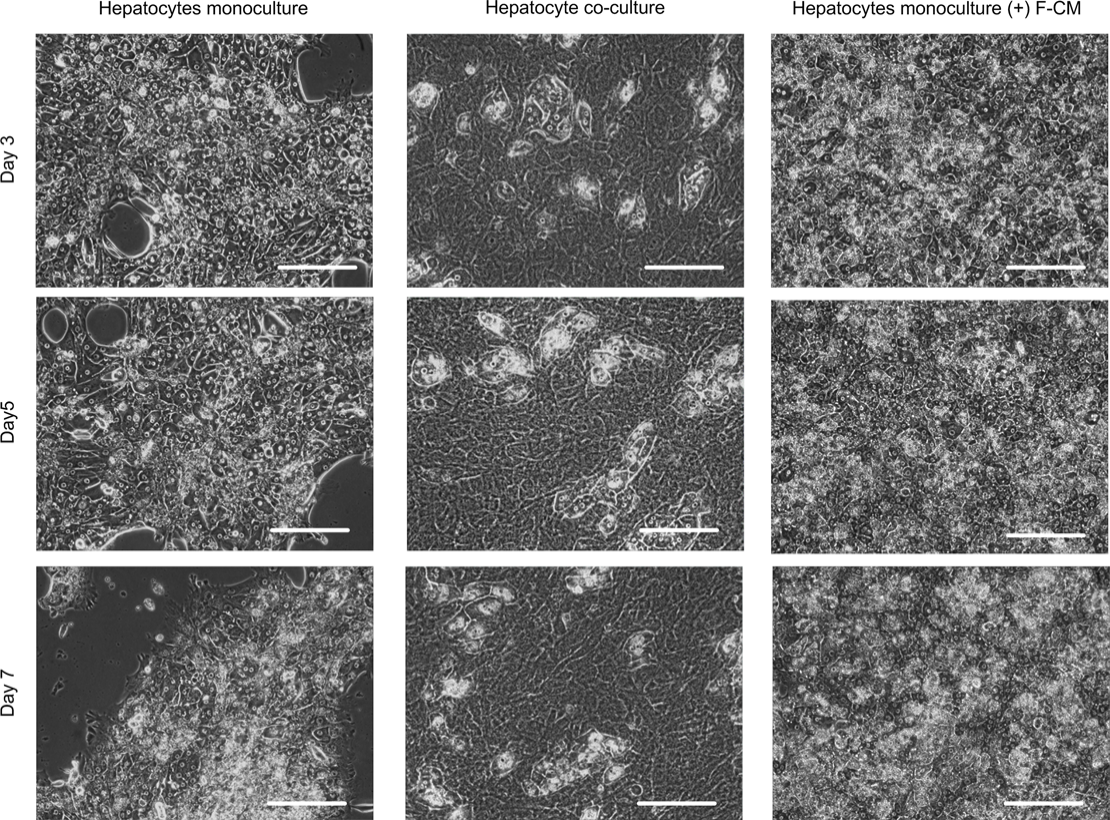
Effect of F-CM on maintaining hepatocyte morphology. Hepatocyte monoculture (left), co-culture with NIH-3T3 J2 (middle) and monoculture with F-CM (55 mg/ml, right) were observed under phase contrast microscope daily for 7 d. Cells with distinct nuclei and bright boundaries are heathy hepatocytes. Scale bars: 200 μm

The morphology of cultured hepatocytes was monitored for 7 d. Hepatocytes without F-CM started to lose their hepatocellular morphology and to detach from the culture plate at day 3. At days 5 and 7, the hepatocytes cultured without F-CM had entirely lost their morphology, and only about 50% of the initial cells were attached to the plate. In contrast, the hepatocyte cultured with concentrated F-CM (55 mg/ml) showed clear hepatocellular morphologies with distinct nuclei and bright cell-cell junctions. This morphology was maintained for 7 d; detachment of the cells was not observed (Fig 3). When concentrated medium (55 mg/ml) that had not been conditioned with fibroblasts was added to the culture instead of F-CM, the degradations in morphology and attachment of cells were more severe than in all other treatments (S1 Fig).

**Fig 4:**
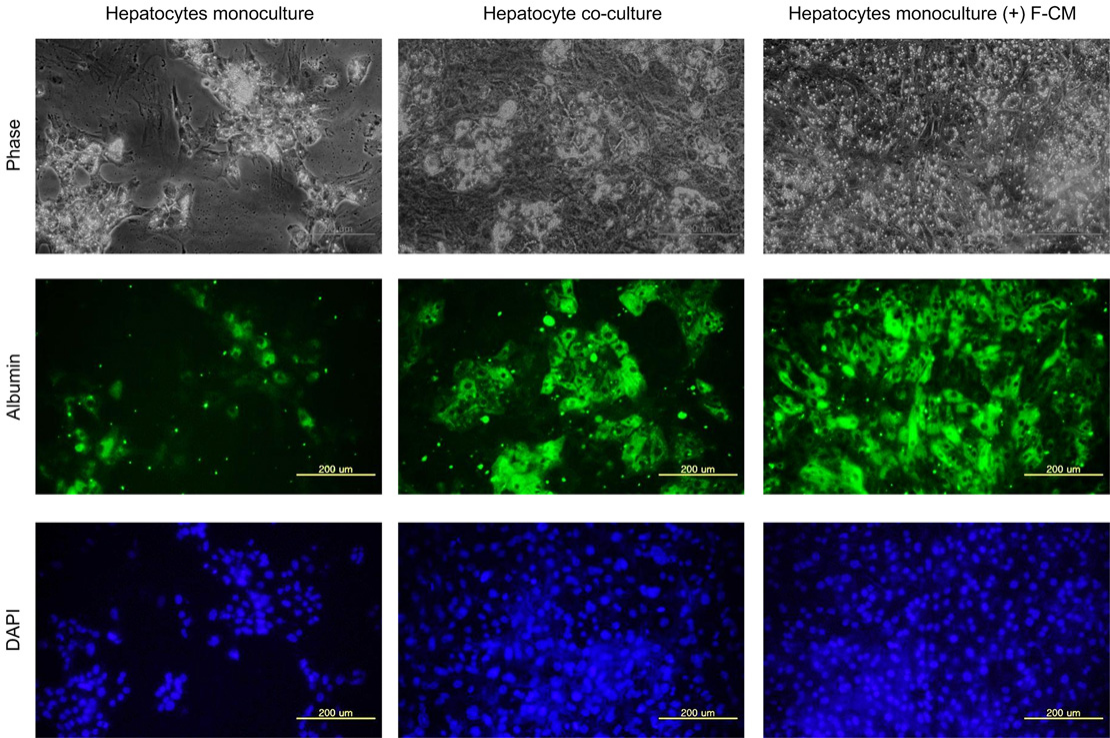
Immuno-histochemistry of intracellular albumin on 9^th^ day of hepatocyte cultures. Hepatocyte monoculture (left), co-culture with NIH-3T3 J2 (middle) and monoculture with F-CM (55 mg/ml, right) on day 9 were fixed and permeabilized using paraformaldehyde and 0.1% Triton X-100, respectively. The fixed samples were then stained with anti-rat albumin primary antibody and Alexa Fluor 488 conjugated secondary antibody (green). Nucleus was counter-stained with DAPI (blue). Scale bars: 200 μm

To examine the supporting effect of F-CM on hepatic functions, intracellular albumin staining was performed using the immuno-fluorescence method. Hepatocytes cultured with concentrated F-CM (55 mg/ml) exhibited intracellular albumin staining that was almost as bright as in the co-culture samples (Fig 4). However, hepatocytes cultured without F-CM showed fewer cells and lower albumin expression than the other two samples.

**Fig 5:**
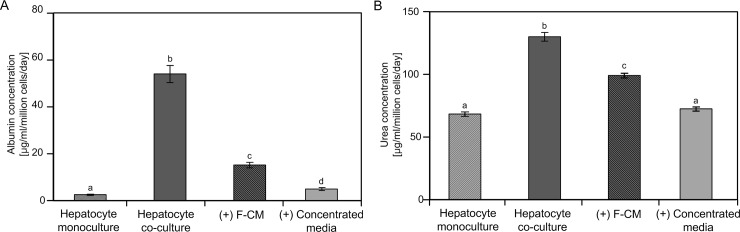
Quantitative analysis of hepatocyte functions. (A) Albumin secretion and (B) urea synthesis of cultured hepatocytes on day 9 were measured using ELISA and colorimetric assay, respectively. “Hepatocyte co-culture” indicates NIH-3T3 J2 co-cultured samples, “(+) F-CM”: hepatocyte monoculture supplemented with F-CM (55 mg/ml); “(+) concentrated media”: hepatocyte monoculture supplemented with concentrated media (unconditioned). Results were analyzed using one-way ANOVA and Tukey’s *post hoc* test. Bars: ± 1 SD, *n* = 9. Bars labeled with different letters (a,b,c and d) are significantly different (Tukey’s test, P<0.01)

Because the morphology and immuno-fluorescence results can provide qualitative information about hepatic functions, quantitative ELISA analyses were further performed to confirm the effect of F-CM on hepatocyte maintenance. One-way ANOVA was conducted to compare the effect of F-CMs on albumin and urea synthesis in mono-culture, co-culture, F-CM and concentrated-media conditions. Both albumin and urea synthesis differed significantly among the four conditions (albumin: F(3, 32) = 257.5, p < 0.001; urea: F(3,31) = 189.3, p < 0.001). *Post hoc* comparisons using Tukey’s test showed that hepatocyte treated with F-CM (55 mg/ml) significantly improved secretion of albumin (M = 15.2, SD = 3.58) and urea (M = 99.2, SD = 5.37) compared to monocultures (albumin: M = 2.54, SD = 0.72; urea: M = 68.4, SD = 4.01) (Fig 5A). Hepatocyte culture with concentrated media (unconditioned) did not significantly affect albumin secretion (M = 4.97, SD = 1.72), but did affect urea secretion (M = 72.5, SD = 4.33) when compared to monocultures (Fig 5B). Although the hepatocytes cultured with F-CM showed increased functions, they were inferior to the function of co-culture case (albumin: M = 54.1, SD = 10.9; urea: M = 99.2, SD = 5.37) (Fig 5). However, hepatocytes cultured with F-CM recovered its albumin function faster than did the co-culture case when albumin and urea secretion were monitored every other day for 9 d (S2 Fig).

**Fig 6:**
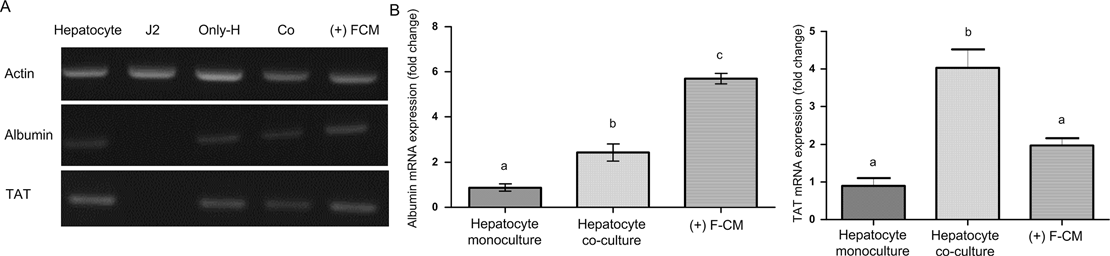
Effects of F-CM on the RNA levels of hepatocytes. (A) PCR for identification of mRNA related to actin, albumin and TAT in hepatocyte; isolated hepatocyte on day 0, J2; NIH-3T3 J2 fibroblast, only-H; hepatocyte monoculture on day 7, Co; Hepatocyte co-culture with NIH-3T3 J2 on day 7, and (+) FCM; hepatocyte monoculture with F-CM (55 mg/ml) on day 7. (B) qPCR for albumin and TAT. the data was analyzed using one-way ANOVA and Tukey’s *post hoc* test. Bars: ± 1 SD, n = 3. Bars labeled with different letters (a,b, and c) are significantly different (Tukey’s test, P<0.01)

To identify the effect of F-CM on hepatocyte, we checked the gene expression levels. For this experiment, hepatocytes were cultured for 7 d in hepatocyte monoculture, hepatocyte co-culture with NIH-3T3 J2, or hepatocyte monoculture with F-CM. After 7 d, all three samples expressed β-actin and liver specific genes, whereas NIH-3T3 J2 fibroblast expressed only β-actin (Fig 6A). Genes for albumin (One-way ANOVA, F(2,6) = 78.56, p<0.001) and tyrosine aminotransferase (TAT) (One-way ANOVA, F(2,6) = 23.386, p<0.01) were expressed 2 and 6 times more, respectively, in the hepatocytes cultured with F-CM (albumin: M = 0.90, SD = 0.31; TAT: M = 5.7, SD = 0.39) than in hepatocyte monocultures (albumin: M = 0.9, SD = 0.39; TAT: M = 2.0, SD = 0.34) (Fig 6B).

**Fig 7:**
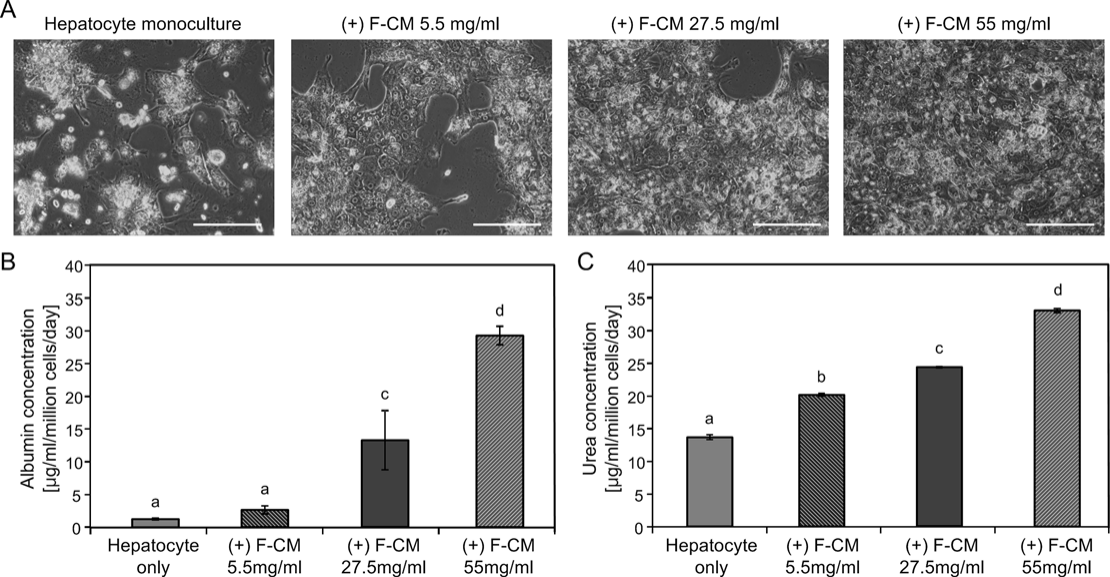
Concentration dependent effects of F-CM on morphology and functions of hepatocytes. Hepatocytes monoculture and monocultures with F-CMs (5.5, 27.5 and 55 mg/ml) were culture for 9 d. (A) Morphologies of one monoculture and three different concentrations of F-CM added samples on day 9 of culture. Scale bars: 200 μm. (B) Albumin secretion and (C) urea synthesis on the same day were measured using ELISA and colorimetric assay, respectively. The results were analyzed using one-way ANOVA and Tukey’s *post hoc* test. Bars: ± 1 SD, *n* > 4. Bars labeled with different letters (a,b,c and d) are significantly different (Tukey’s test, p<0.01)

To confirm that the hepatic functions were improved by F-CM-supplemented media, the effects of different concentrations of F-CM in hepatocyte culture were measured. As the concentration of F-CM in culture media increased, the morphology and attachment of hepatocytes improved (Fig 7A). On day 9 approximately half of the hepatocytes were detached both in the no-CM and low-concentration F-CM media (5.5 mg/ml), but almost all seeded hepatocytes were attached in the high-concentration F-CM media (55 mg/ml). A similar tendency in hepatic function was observed as measured by secreted albumin and urea ELISA. Albumin secretion increased as the concentration of F-CM media increased, but low concentration of F-CM media (5.5 mg/ml) could not notably increase the albumin secretion on day 9 (One-way ANOVA, F(3,14) = 560, p < 0.001). Urea synthesis also increased as the concentration of F-CM treatment increased and even the low-concentration F-CM media (5.5 mg/ml) increased urea synthesis by hepatocytes on day 9 (One-way ANOVA, F(3,11) = 3323, p<0.001).

## Discussion

This study explored the effects of F-CM on *in vitro* primary rat hepatocyte culture. Albumin secretion and urea cycling are liver specific markers that represent the protein synthesis and metabolic functions of the liver, respectively. TAT is a liver-specific gene that codes for an enzyme, and is also marker for hepatocyte differentiation. Quantifying the level of cell-specific protein is one of the most general methods for measuring the functional activities of cells. Therefore, measuring the level of albumin secretion has been commonly used as a method in hepatocyte research because albumin is the most abundant protein among other hepatocyte-secreting factors. [30,31]

Metabolic functions such as the urea cycle are important functions of hepatocytes. Thus, measuring the amount of urea converted by hepatocytes is a critical indicator of metabolic function of hepatocytes. [31,32]. Although other factors can also represent hepatic functions, the assessment of just these two markers can provide enough information to evaluate the condition of cultured hepatocytes.

Confirmation of gene expression levels is a general method to check the functional activity of cells and to characterize them. In this study, PCR demonstrated that hepatocytes in all the conditions expressed liver-specific genes. According to qPCR, albumin mRNA expression levels in hepatocyte increased in the presence of F-CM. TAT mRNA expression levels in hepatocyte cultured with F-CM were not distinguished from in hepatocyte monocultures by the one-way ANOVA and Tukey’s *post hoc* test. However, TAT mRNA expression levels in three conditions were different by one-way ANOVA test (p<0.05). This may also be a meaningful result. Extension of culture time might result in increase in the difference of gene expression. When hepatocytes are cultured with F-CM, hepatocyte functions and liver specific gene expression can be maintained.

The viability and functions (albumin secretion and urea cycle) of hepatocytes treated with concentrated F-CM were comparable to those of hepatocytes co-cultured with fibroblasts. Because the group treated with concentrated medium (unconditioned) did not show such effects, the fibroblast-secreted factors in F-CM might be responsible for the increases in hepatocyte functions. The concentration-dependent improvement of hepatic functions by F-CM treatment is also consistent with the observation that F-CM contains some factors that can sustain *in vitro* hepatocyte cultures.

SEC analysis of F-CM and concentrated medium revealed some unmatched peaks that indicate the factors produced or consumed by the fibroblasts. Fibroblasts secrete various factors such as extracellular matrix proteins (e.g., collagen I, decorin) and cytokines (e.g., interleukin-1, interleukin-6, vascular endothelial growth factor (VEGF)). Among these factors, we selected some that are considered to be related to the functions of primary hepatocyte and that have sizes that correspond to peaks revealed from SEC analysis. The most probable candidate factor for the peak near 45 kDa is VEGF (34~42 kDa) (Fig 2). VEGF stimulates secretion of other growth factors, and protects the liver against toxic damage. [33] Gene expression profile of VEGF, especially VEGF-D, correlates positively with induction of liver-specific functions. [26]

Because concentrated media with serum have too many proteins, which can clog the column and may interfere with the protein signals of fibroblast secretions, an additional SEC analysis was performed using concentrated cultured serum-free (SF) media and concentrated SF media (S3 Fig). One broad peak (17~24 min) was detected; the size of the corresponding protein is approximately 40~500 kDa. One probable candidate protein for this peak is pro-collagen (100~ 300 kDa), which might be synthesized to collagen in high concentration of F-CM. Collagen is an essential ECM protein for hepatocyte attachment. [34,35] This peak is also compatible with decorin (90~140 kDa) which is also a proteoglycan; it binds to collagen and is strongly upregulated during liver regeneration [6].

In previous studies, both matrix deposition and direct cell-cell contact were identified as the most important phenomena that maintain hepatocyte functions in co-cultures, whereas soluble factors are generally shown to be ineffective [18]. However, in contrast to these findings, the present study showed that the highly-concentrated soluble factors secreted by fibroblasts can significantly improve the functions of hepatocytes to levels similar to those in hepatocytes co-cultured with fibroblasts; this result contradicts previous results that suggested that cell-cell contact is necessary for *in vitro* maintenance of hepatocyte structure and function.

The major difference of this work from previous studies that used conditioned media is that in this work a process was used to concentrate the fibroblast-conditioned medium. Using this concentrated medium, we clearly demonstrated that soluble factors can support hepatic functions and viability without direct cell-cell contact in *in vitro* hepatocyte cultures; the previous studies that did not observe a co-culture effect in the absence of physical contact may have used insufficient amount or concentration of factors. We do not yet know whether such factors exist in F-CM, and if they exist, how they affect hepatocytes. If we find certain factors in further research, the findings can improve our understanding of the mechanism of hepatocyte co-culture. It also can provide a simple and efficient method to culture hepatocytes *in vitro*.

## Supporting Information

S1 Fig Attachment of hepatocytes on 7^th^ day of culture. “Hepatocytes co-culture”: NIH-3T3 J2 co-cultured samples, “(+) F-CM”: hepatocyte monoculture supplemented with F-CM (55 mg/ml), “(+) concentrated media”: hepatocyte monoculture supplemented with concentrated media (unconditioned). Scale bars: 200 μm.
(TIF)

S2 Fig Monitoring of hepatocyte functions bi-daily. (A) Albumin secretion and (B) urea synthesis of cultured hepatocytes were monitored every other day for 9 days using ELISA and colorimetric assay, respectively. “Hepatocytes co-culture”: NIH-3T3 J2 co-cultured samples, “(+) F-CM”: hepatocyte monoculture supplemented with F-CM (55 mg/ml). Bars: ± 1 SD, *n* = 3.
(TIF)

S3 Fig size exclusion chromatography result of cultured serum free condensed media and non-cultured serum free media. Qualitative composition of concentrated cultured serum free media (orange dot line) and concentrated non-cultured serum free media (sky blue dot line) were analyzed using size-exclusion chromatography. Black line: composition of standard protein to indicate reference size.
(TIF)
