# Supplementary figures and images for: Effect of Concentrated Fibroblast-Conditioned Media on In Vitro Maintenance of Rat Primary Hepatocyte
Source: PLoS One. 2016 Feb 10;11(2):e0148846. doi: 10.1371/journal.pone.0148846 (PMC4749383; doi:10.1371/journal.pone.0148846)

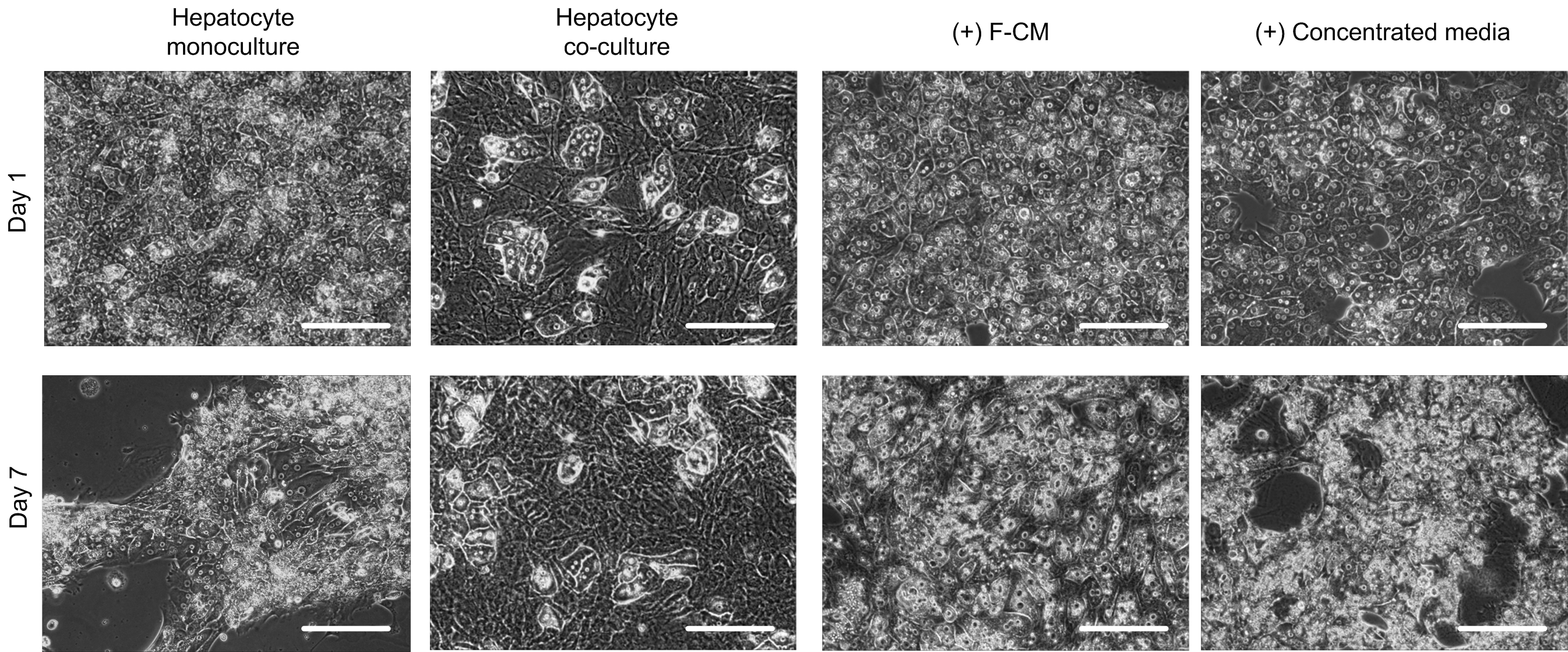

Supplement: S1 Fig — “Hepatocytes co-culture”: NIH-3T3 J2 co-cultured samples, “(+) F-CM”: hepatocyte monoculture supplemented with F-CM (55 mg/ml), “(+) concentrated media”: hepatocyte monoculture supplemented with concentrated media (unconditioned). Scale bars: 200 μm. (TIF) [file pone.0148846.s001.tif]

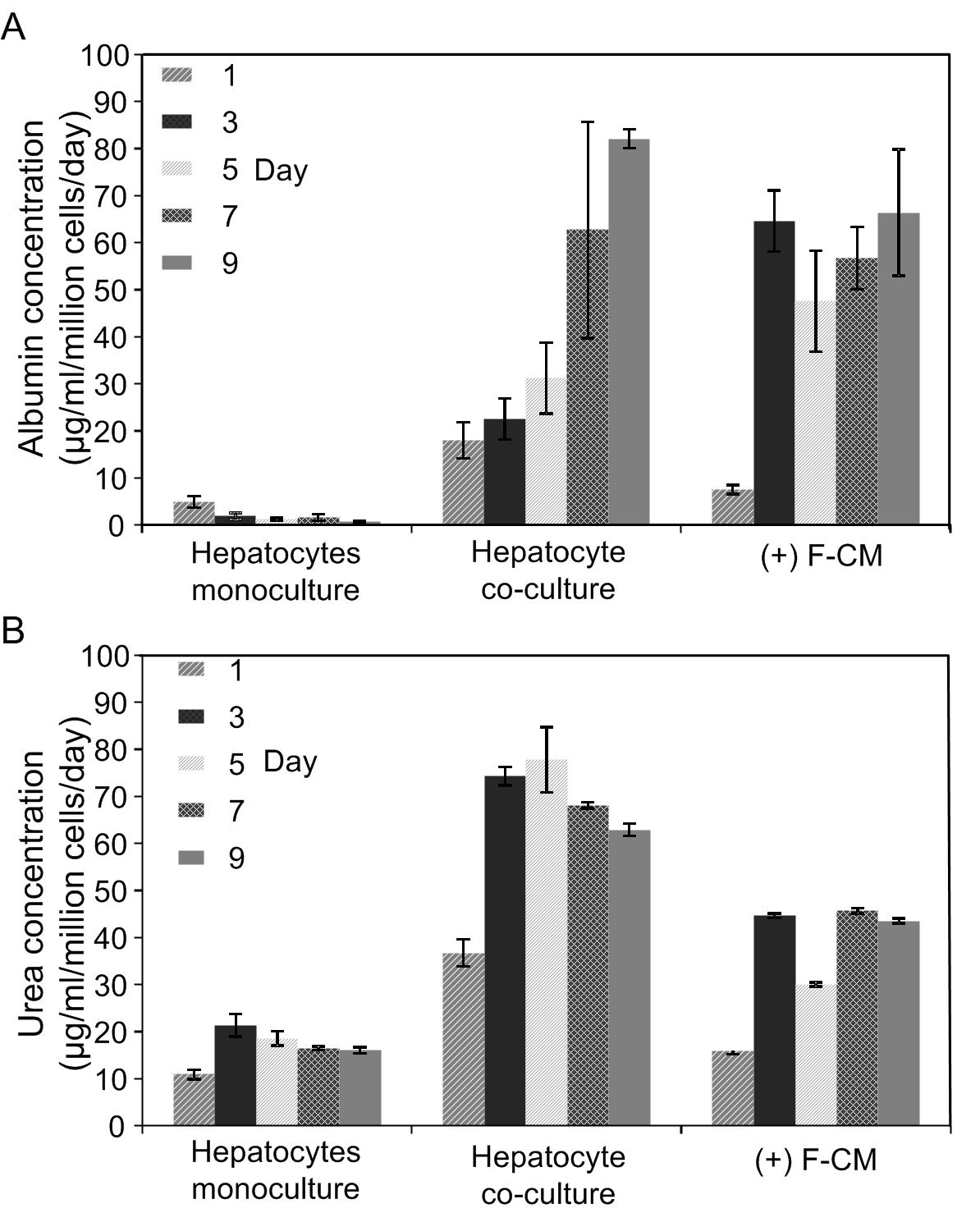

Supplement: S2 Fig — (A) Albumin secretion and (B) urea synthesis of cultured hepatocytes were monitored every other day for 9 days using ELISA and colorimetric assay, respectively. “Hepatocytes co-culture”: NIH-3T3 J2 co-cultured samples, “(+) F-CM”: hepatocyte monoculture supplemented with F-CM (55 mg/ml). Bars: ± 1 SD, n = 3. (TIF) [file pone.0148846.s002.tif]

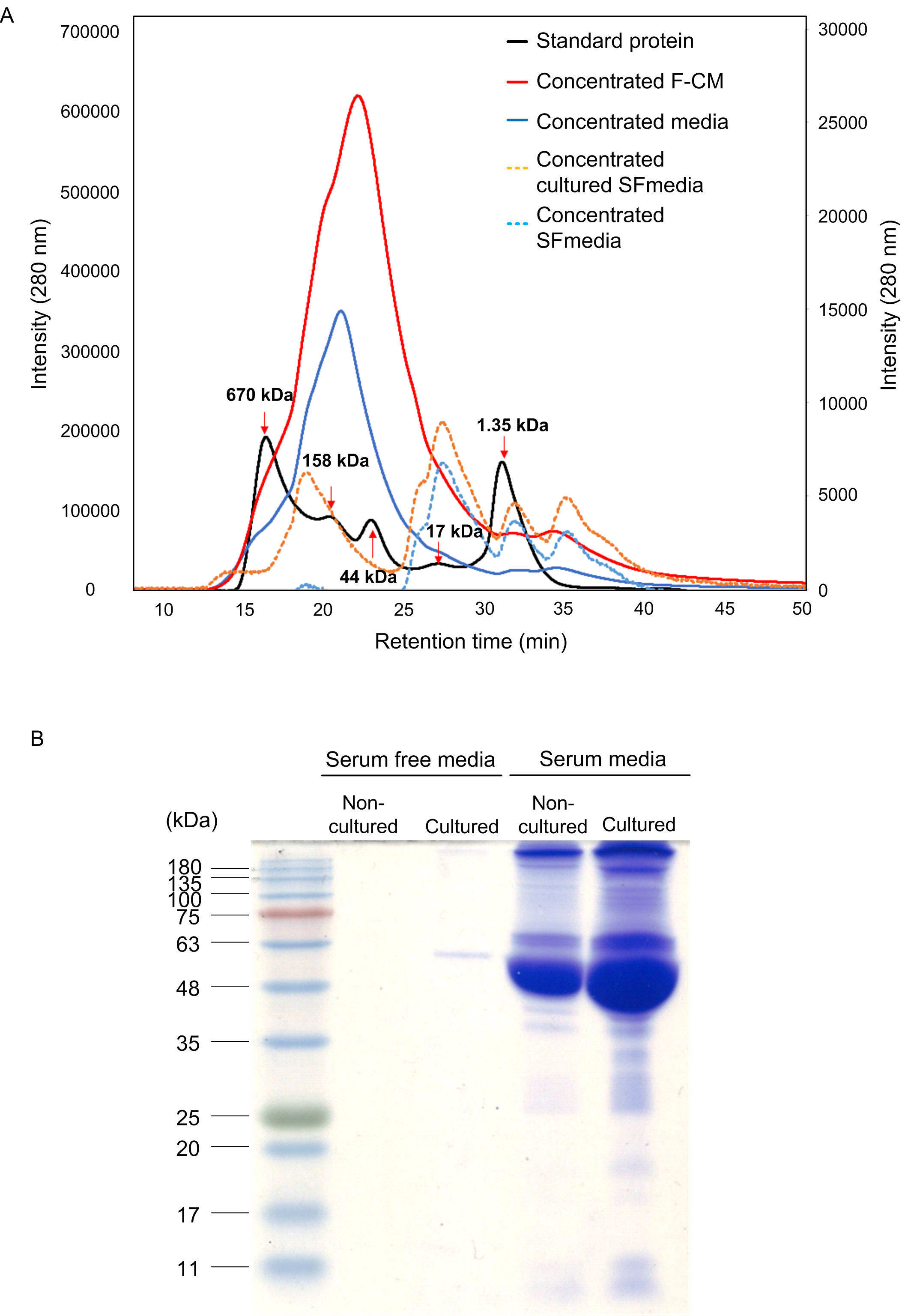

Supplement: S3 Fig — Qualitative composition of concentrated cultured serum free media (orange dot line) and concentrated non-cultured serum free media (sky blue dot line) were analyzed using size-exclusion chromatography. Black line: composition of standard protein to indicate reference size. (TIF) [file pone.0148846.s003.tif]
